# Bournemouth as a Health Resort

**Published:** 1889-06

**Authors:** 


*Bournemouth as a Health Resort.* By A. KINSEY-MORGAN, M.R.C.S. Pp. 98. London: SIMPKIN, MARSHALL and Co. 1889.

This is a pleasantly written, abundantly illustrated and thoroughly satisfactory medical guide to one of our best and most popular home health-resorts. The usual geological, hydrographic, meteorological and scenic material is provided with judiciousness and not too elaborately. We could have put up with a little more help towards the selection of cases likely to be benefited by a residence at Bournemouth, and we might easily dispense with the somewhat lengthy account of the disabilities of foreign health-resorts. Bournemouth as a health-resort is one thing; other places as failures in this respect is another and different thing, and can scarcely be properly treated under the former title. True, from both personal and vicarious experience, we most heartily sympathise with the author. We have over-estimated the value of foreign health-resorts and under-estimated the value of home ones. We are too liable to look only to average temperature, or elevation, or sunshine, or sea-air, and so forth, and entirely overlook the thousand and one adjuncts which are essential to the health and comfort of a civilised Briton. It may be true that on certain table-lands and mountains and islands semi-civilised or wholly savage Africans, or Americans, or Australians never have phthisis; it is a late, and often a lame, conclusion to infer that the cultured and refined Britisher who is consumptive may cure himself by reverting to this primitive type and living this life. The balmy, sunny, marine, sheltered and resinous climate of Bournemouth, with its home comforts and its sanitary wholesomeness, cannot easily be surpassed as a means of restoring health in many complaints.

